# Regulation of cardiac cellular bioenergetics: mechanisms and consequences

**DOI:** 10.14814/phy2.12464

**Published:** 2015-07-30

**Authors:** Kenneth Tran, Denis S Loiselle, Edmund J Crampin

**Affiliations:** 1 Auckland Bioengineering Institute, University of AucklandAuckland, New Zealand; 2 Department of Physiology, University of AucklandAuckland, New Zealand; 3 Systems Biology Laboratory, Melbourne School of Engineering, University of MelbourneParkville, Victoria, Australia; 4 School of Mathematics and Statistics, University of MelbourneParkville, Victoria, Australia; 5 School of Medicine, University of MelbourneParkville, Victoria, Australia

**Keywords:** Cardiac cellular bioenergetics, cell model, mitochondrial regulation

## Abstract

The regulation of cardiac cellular bioenergetics is critical for maintaining normal cell function, yet the nature of this regulation is not fully understood. Different mechanisms have been proposed to explain how mitochondrial ATP production is regulated to match changing cellular energy demand while metabolite concentrations are maintained. We have developed an integrated mathematical model of cardiac cellular bioenergetics, electrophysiology, and mechanics to test whether stimulation of the dehydrogenase flux by Ca^2+^ or Pi, or stimulation of complex III by Pi can increase the rate of mitochondrial ATP production above that determined by substrate availability (ADP and Pi). Using the model, we show that, under physiological conditions the rate of mitochondrial ATP production can match varying demand through substrate availability alone; that ATP production rate is not limited by the supply of reducing equivalents in the form of NADH, as a result of Ca^2+^ or Pi activation of the dehydrogenases; and that ATP production rate is sensitive to feedback activation of complex III by Pi. We then investigate the mechanistic implications on cytosolic ion homeostasis and force production by simulating the concentrations of cytosolic Ca^2+^, Na^+^ and K^+^, and activity of the key ATPases, SERCA pump, Na^+^/K^+^ pump and actin-myosin ATPase, in response to increasing cellular energy demand. We find that feedback regulation of mitochondrial complex III by Pi improves the coupling between energy demand and mitochondrial ATP production and stabilizes cytosolic ADP and Pi concentrations. This subsequently leads to stabilized cytosolic ionic concentrations and consequentially reduced energetic cost from cellular ATPases.

## Introduction

Cardiac myocytes utilize the free energy of ATP hydrolysis (ΔG_ATP_) to drive key cellular processes responsible for force development and cellular ion homeostasis. The dominant cellular consumers of ATP are the actin-myosin ATPase, the SERCA pump, and the Na^+^/K^+^ ATPase (Na pump). Mitochondria maintain cytosolic ΔG_ATP,_ in response to changing cellular energy demand by resynthesizing ATP from its hydrolysis products ADP and Pi via oxidative phosphorylation. Given the requirement for a heart to continuously pump without rest, changes in energy demand must be quickly matched by supply. When transitioning from a low to a high workload, the myocyte can match the rate of mitochondrial ATP production to a several-fold increase in ATP utilization with little or no observed changes in the concentrations of metabolite intermediates (Balaban et al. 1986). This regulation is both remarkable and of paramount importance to the normal functioning of the heart, and is a central question of cardiac physiology that is yet to be fully understood.

On the basis of metabolite stability, a feed-forward mechanism centered on Ca^2+^ as a signaling messenger was postulated (Balaban 2002). The theory that Ca^2+^ plays a crucial role in regulating and maintaining cellular metabolic homeostasis is an appealing one, given the ubiquitous presence of Ca^2+^ throughout the myocyte and its functional role in activating energy-demanding cellular processes. The large Ca^2+^ gradients between the cytosol, extracellular space, and various compartmentalized spaces are a store of potential energy driven by ΔG_ATP_ which enable large and rapid changes in local Ca^2+^ concentrations required for initiating many signaling pathways including regulation of mitochondrial energy supply (Glancy and Balaban 2012). A number of studies have demonstrated stimulatory effects of mitochondrial Ca^2+^ on various matrix dehydrogenases (Denton et al. 1978; Kobayashi and Neely 1983; Hansford 1987; McCormack et al. 1990; Harris and Das 1991). However, these stimulatory effects have been shown to be completely saturated at the lowest physiological Ca^2+^ concentrations under physiological temperature and ionic conditions (Vinnakota et al. 2011).

More recently, a competing hypothesis proffered by Beard and colleagues (Wu et al. 2008; Beard and Kushmerick 2009) proposed a mechanism whereby inorganic phosphate-mediated feedback alone is sufficient for cardiac energetic regulation, in contradistinction to the metabolic stability theory of Balaban et al. (1986). The hypothesis was based on an in silico study (Beard 2005) where, out of 19 other possible mechanisms, only inorganic phosphate regulation of mitochondrial respiratory complex III was capable of improving the fit of the mitochondria model to data on isolated mitochondria from Bose et al. (2003). Using comprehensive models of the respiratory system and oxidative phosphorylation (Wu et al. 2008, 2009) with Pi-dependent feedback of mitochondrial respiratory complex III, they were able to reproduce in vivo whole heart experimental data relating the rate of oxygen consumption to metabolite concentrations and to predict an increase in cytosolic Pi with increasing work load in the absence of any Ca^2+^-mediated mechanisms.

There are, thus, discordant findings in the literature and no consensus view as to what determines how mitochondrial ATP production varies to match changing demand in the cardiomyocyte. Specifically, one school of thought argues that metabolite levels do not change sufficiently with changing work load to regulate mitochondrial ATP production, and proposes a role for calcium ions in mediating this feedback; while the other argues that metabolite concentrations do indeed change sufficiently to regulate ATP production, and specifically proposes a role for inorganic phosphate in mediating this energetic coupling.

In this study, we use mathematical modeling to investigate the significance of Ca^2+^-mediated feed-forward versus Pi-mediated feedback mechanisms in regulating energy supply to match energy demand. A number of in silico studies have yielded contrasting conclusions with some concluding that Pi and ADP feedback is insufficient (Cortassa et al. 2003; Jo et al. 2006; Korzeniewski 2006) and others concluding otherwise (Vendelin et al. 2000; Wu et al. 2008, 2009). These studies are centered on well-defined mitochondrial models which are driven by very simplistic, empirically-based models of cellular energy demand which do not account fully for the dependence of ATP consumption rate on ATP, ADP and Pi concentrations. Although the demand sides of these models consist of ATPases that are driven by ATP hydrolysis, their kinetics are insensitive to both the cytosolic concentrations of ATP and its hydrolysis products: ADP and Pi. The overall function of the myocyte is determined by how well the regulatory mechanism matches energy supply to energy demand to maintain metabolite and cellular ion homeostasis. We have therefore developed an integrated model of cardiac bioenergetics, electrophysiology, and cellular mechanics, which couples thermodynamic models of metabolite-sensitive energy-demand processes to mitochondrial energy supply. The model demonstrates: (1) that the availability of substrates, ADP and Pi, in the absence of any other feedback mechanism can couple energy supply to changing demand; (2) that modulation of the dehydrogenase flux through activation by Ca^2+^ or Pi is unlikely to increase mitochondrial ATP supply for a given level of energy demand; and (3) that Pi-activation of complex III may improve metabolic stability above that provided by substrate availability feedback. Finally we use the model to determine the implications of these findings for cardiomyocyte ion homeostasis in the context of the overall energetic cost to the cell over the cardiac cycle.

## Methods

**Figure 1:**
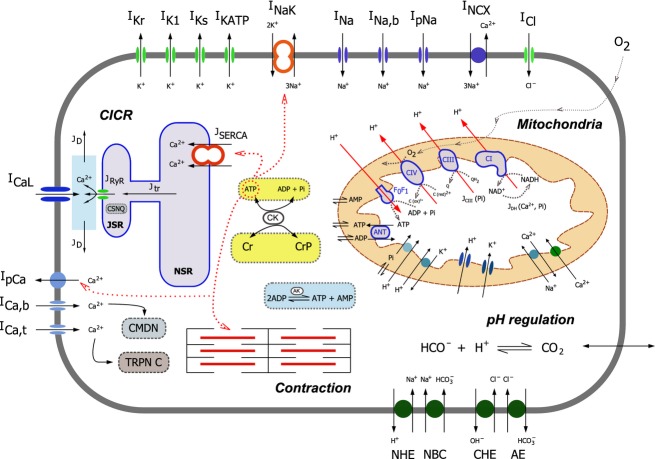
A schematic of cell model. The regulatory mechanisms investigated in this study are the regulation of the dehydrogenase flux (J_DH_) by Ca^2+^ and Pi and the regulation of complex III by Pi (J_CIII_).

The ventricular myocyte cell model used in this study is based on that of Crampin and Smith (2006). It builds on the guinea pig electrophysiology model of Faber and Rudy (2000) by integrating tension development as well as incorporating pH dependence into processes involved in intracellular Ca^2+^ homeostasis. Four acid/base transporters were added to model the regulation of intracellular proton concentration. Consistent with the model of Faber and Rudy (2000), guinea pig data were used to validate the four acid/base transporters. The electrophysiological properties of the whole-cell model are described by ion fluxes through ion-specific channels and transporters and the concentrations of these ions are tracked by solving a coupled system of nonlinear ordinary differential equations (ODEs). The following outlines the key features of the myocyte model presented as a schematic in Figure1.

### ATP consumption

The three major ATP-consuming processes in the myocyte are the Na^+^/K^+^ pump, the SERCA pump and cross-bridge cycling. In the Crampin and Smith (2006) model, these ATPases are insensitive to changes in metabolite concentrations and therefore incapable of simulating events where the energetic state of the cell may be changing. These have been replaced by more recent models which have been developed specifically to address the issue of metabolite sensitivity. These are described below.

#### Na^+^/K^+^ pump

The Na^+^/K^+^ pump model is replaced by that described in Terkildsen et al. (2007), which uses the same Na^+^ and K^+^ data set as that of Faber and Rudy (2000) but, in addition, also captures the response of the pump to changes in metabolite concentrations. The pump is modeled as a four-state enzymatic cycle and is subject to the usual constraints to satisfy thermodynamic consistency.

#### SERCA

Following the methods outlined by Smith and Crampin (2004) in the development of the Na^+^/K^+^ pump, we have developed a model of SERCA (Tran et al. 2009) for the purposes of simulating cardiac energetics. The importance of incorporating thermodynamic cycle constraints in the model was highlighted by the investigations of the study into the reversibility of the SERCA cycle (Loiselle et al. 2010).

#### Cross-bridge cycling

The model of tension development is replaced with the cross-bridge model from Tran et al. (2010). This is based on the Rice et al. (2008) cross-bridge model which describes the Ca^2+^ activation and cross-bridge cycling kinetics of cardiac muscle using a mean field ODE formulation. The Tran et al. (2010) model builds on this while preserving the original properties of the model, by introducing metabolite-binding steps as well as imposing thermodynamic constraints on the cycle.

### Mitochondrial ATP supply

We integrated the Beard (2005) model of mitochondrial ATP production into the cell model to describe the synthesis of ATP via oxidative phosphorylation. The parameterization of the model is based on the data from isolated mitochondria which capture the relationships between metabolite levels, oxygen flux and work rate (Bose et al. 2003). The model does not explicitly include the tricarboxylic acid (TCA) cycle and other NADH-producing reactions but instead, uses a phenomenological driving force to simulate an overall phosphate-dependent dehydrogenase flux reaction: 

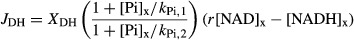
1where [Pi]_x_ is the concentration of inorganic phosphate in the mitochondrial matrix compartment. The definitions of the other parameters are given in Beard (2005) (eq. 1). The equation describes a feedback mechanism where inorganic phosphate can upregulate the dehydrogenase flux in response to an increase in energy demand. This is one of three mechanisms that will be tested below. Within the respiratory chain, the mitochondrial model also has inorganic phosphate feedback modulating the activity of complex III: 

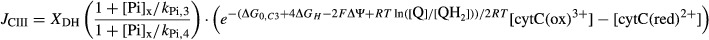
2

This mechanism was required to adequately fit a model of isolated mitochondria to experimental data on membrane potential (Beard 2005) (eq. 24). This is the second of the three mechanisms which will be tested.

### Mitochondrial Ca uniporter and Na^+^/Ca^2+^ exchanger

The third mechanism that is to be tested is the feedback of mitochondrial Ca^2+^ onto intermediates within the TCA cycle. To implement this feedback, models of the mitochondrial Ca^2+^ uniporter and Na^+^/Ca^2+^ exchanger (Cortassa et al. 2003) were integrated into the mitochondria model. In the absence of a TCA cycle model, the effect of mitochondrial Ca^2+^ was modeled as a modifier of the dehydrogenase flux, the rationale being that the ultimate effect of mitochondrial Ca^2+^ on the various matrix dehydrogenases is to regulate the dehydrogenase flux.

To capture the dependence of the dehydrogenase flux on mitochondrial Ca^2+^ concentration, equation 1 was modified as follows: 

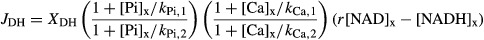
3

**Figure 2:**
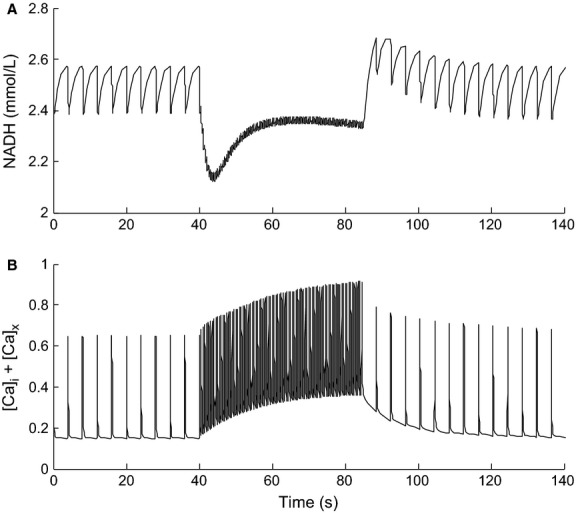
Model simulation whereby the pacing frequency of the cell model was increased from 0.25 to 2 Hz at *t* = 40 sec and reduced back to 0.25 Hz at *t* = 85 sec. The top panel shows the response of the NADH concentration and the bottom panel shows the response of the total free Ca^2+^ in the cell.

The two parameters governing the mitochondrial Ca^2+^ dependence were constrained by fitting the model to data on the time course of NADH and cytosolic Ca^2+^ concentrations in response to an increase in the stimulus frequency from 0.25 to 2 Hz in rat cardiac trabeculae (Brandes and Bers 2002) (Fig.2). In order to obtain a reasonable fit to the data, the original value of *X*_DH_ was also reduced as its large magnitude kept NADH at saturating levels, preventing it from responding to changes in pacing frequency.

### Calculation of ATP consumption rate

It is assumed that the hydrolysis of magnesium-bound ATP (MgATP) is tightly (stoichiometrically) coupled to the enzymatic cycles of each of the three ATPases; i.e., for each cycle, one ATP molecule is consumed followed by the release of one magnesium-bound ADP (MgADP), one inorganic phosphate (Pi) and one proton. The SERCA and Na^+^/K^+^ pumps are modeled as steady-state cycles which allow the calculation of an overall cycle rate. This rate is proportional to the rate of ATP hydrolysis. The cross-bridge cycle, on the other hand, is not modeled as a steady-state cycle because the dynamic aspects of the cross-bridge cycle are fundamental to producing the cardiac force transient. The ATP consumption rate is obtained by calculating the net flux from state *XB*_PostR_ to state *P*_XB_ (Tran et al. 2010) as this reaction step is responsible for the binding and hydrolysis of MgATP: 

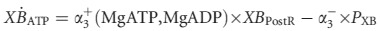
4

Energy consumption in the model is quantified by the rate of ATP consumption. Full details of the cell model are available from the author.

### Simulation protocol

#### Frequency change simulations

The cell model was stimulated under isometric conditions at a pacing frequency of 2 Hz and a sarcomere length (SL) of 2.1 *μ*m until a beat-to-beat steady-state was reached. The energy demand was raised by increasing the stimulus frequency to 3 Hz for approximately 3 min before reducing back to 2 Hz again. The simulation protocol is repeated for the following cases.

Model 1: Substrate (ADP and Pi) availability only (none of the other feedback mechanisms are active in the model).
Model 2: Substrate availability and mitochondrial Ca^2+^ feedback on dehydrogenase flux.
Model 3: Substrate availability and Pi feedback on dehydrogenase flux.
Model 4: Substrate availability and Pi feedback on mitochondrial complex III.
Model 5: Substrate availability and all three additional mechanisms are active.

Underlying these protocols is the substrate availability feedback pathway which is an intrinsic property of the model, arising from the coupling of the mitochondria to the rest of the cell model. The metabolites ADP and Pi are both the by-products of ATP hydrolysis, and natural substrates for mitochondrial ATP regeneration. Hence, ADP and Pi levels provide an intrinsic feedback on ATP production rate.

To inactivate each of the three feedback mechanisms (mitochondrial Ca^2+^ regulation of dehydrogenase flux; Pi regulation of the dehydrogenase flux; Pi regulation of mitochondrial complex III), the value of mitochondrial matrix Pi ([Pi]_x_), or mitochondrial matrix Ca^2+^ ([Ca]_x_) in the modifier term in the relevant equation (eqs 1,2,3) was kept constant at the steady-state beat-to-beat value reached under 2 Hz stimulus, before increasing the frequency to 3 Hz.

#### Length change simulations

The length change protocol was adopted to investigate the effect of the three mitochondrial regulatory mechanisms on cellular ion homeostasis and force production. Using an iso-frequency approach, along with removal of the effects of length-dependent binding of Ca^2+^ to troponin on the intracellular Ca^2+^ pool, allowed us to attribute any observed changes in Ca^2+^ concentration to be driven by mitochondrial regulation. For these simulations, the cell model was stimulated for 7 min at 3 Hz, at SLs ranging from 1.6 *μ*m to 2.1 *μ*m, until a steady-state was reached. The protocol was repeated for the five cases outlined above.

## Results

### Cell model validation

We simulated isometric contractions at a stimulation frequency of 2 Hz until a stable beat-to-beat steady-state was reached. Table1 shows the average steady-state metabolite and ion concentrations generated by the simulation. The concentrations of these metabolites are variables in the model, and hence these steady-state values are determined by the dynamic feedback between energy demand and energy supply. This provides a good test of the coupling between the excitation-contraction processes and mitochondrial ATP production within the model.

**Table 1:** Average steady-state values of the cytosolic metabolite and ion concentrations in the cell model at a stimulation frequency of 2 Hz and SL = 2.1 *μ*m

Variable	Value	Units
[MgATP]_i_	5.61	mmol L^−1^
[MgADP]_i_	25.4	*μ*mol L^−1^
[Pi]_i_	1.16	mmol L^−1^
[PCr]_i_	20.5	mmol L^−1^
pH_i_	7.15	Unitless
[Na^+^]_i_	9.6	mmol L^−1^
[K^+^]_i_	130	mmol L^−1^

To validate the ATPase rates predicted by the model, we compare the output of the model to data from Schramm et al. (1994) who measured the *active* rate of heat production in guinea pig ventricular myocytes at 37°C in order to determine the relative contributions of the three main ATPases in the cell. The active rate is calculated by subtracting the basal rate from the total ATP consumption rate. The ATP consumption rates from cross-bridge cycling and from the SERCA pump are pulsatile due to the transient nature of the Ca^2+^ signal whereas the ATP consumption rate from the Na^+^/K^+^ pump remains relatively constant. The variation in the cycling rate of the Na^+^/K^+^ pump is small because of the necessarily high basal rate required to maintain the negative membrane potential due to Na^+^ and K^+^ leakage currents. During diastole, the rate of energy consumption from cross-bridge cycling at 2 Hz is negligible while that of the SERCA pump is very small. This very small SERCA flux is required to counteract the leak current from the SR.

To compare the relative contributions of each of the three ATPases to total energy consumption, an average ATP consumption rate can be calculated by integrating over a beat and dividing by the period (Table2). Cross-bridge cycling is responsible for the majority of energy consumption over a beat (85%), followed by SERCA (10%) and the Na^+^/K^+^ (5%) pump. Quantitatively, the total ATP consumption rates from the model are in good agreement with the experimental literature; however, the cross-bridge cycle in the model makes up a slightly larger proportion of the total energy consumption rate than is experimentally observed. This, along with the physiological steady-state metabolite and ion concentration values generated by the model (Table1), provides important quantitative validation of the model.

**Table 2:** Comparison of average ATPase consumption rates from the whole-cell model to data from guinea pig trabeculae (Schramm et al. 1994). The relative contributions (%) of each of the ATPases are given in parentheses. The model values were obtained at the same stimulation frequency as the experimental data (2 Hz) with SL = 2.1 *μ*m

	Na^+^/K^+^ (mmol L^−1^ ms^−1^)	SERCA (mmol L^−1^ ms^−1^)	Cross-bridge (mmol L^−1^ ms^−1^)	Total (mmol L^−1^ ms^−1^)
Model	2.84 × 10^−5^ (5)	5.60 × 10^−5^ (10)	5.01 × 10^−4^ (85)	0.585 × 10^−3^
Data	4.96 × 10^−5^ (9)	8.27 × 10^−5^ (15)	4.20 × 10^−4^ (76)	0.551 × 10^−3^

### Effects of the different postulated feedback mechanisms on regulating cytosolic metabolite concentrations

**Figure 3:**
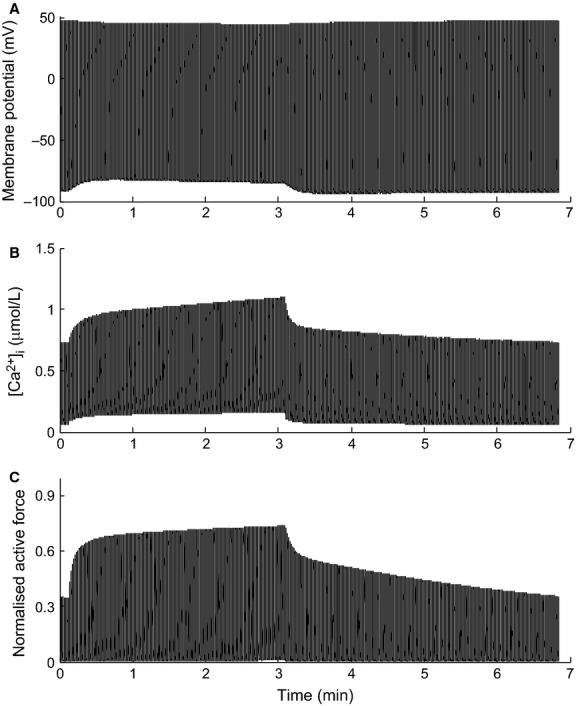
Cell model simulation showing the response of the sarcolemmal membrane potential (A), intracellular Ca^2+^ concentration (B) and active force production (C) to an increase of frequency. The frequency was increased from 2 Hz to 3 Hz after *t* = 15 sec, and reduced back to 2 Hz at *t* = 3 min. The simulation was run at 2 Hz until a beat-to-beat steady-state was reached before increasing to 3 Hz. All three mitochondrial regulatory mechanisms are active in the simulation.

**Figure 4:**
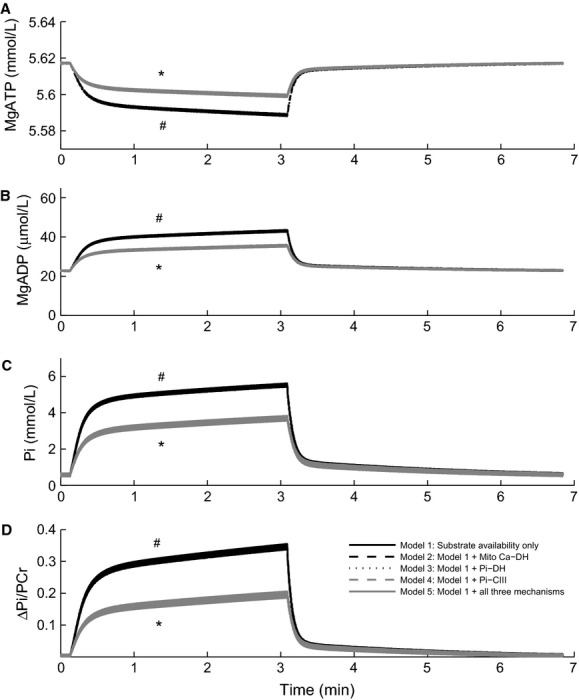
Cell model simulation of the effect of activating different feedback mechanisms on the cytosolic concentrations of MgATP (A), MgADP (B), Pi (C) and ΔP/PCr (D) in response to a change of stimulus frequency from 2 Hz to 3 Hz at *t* = 15 sec and back to 2 Hz at *t* = 3 min. During the 2 Hz simulation period, all three mitochondrial regulatory mechanisms were active. ^#^Models 1, 2 and 3 are superimposed. *Models 4 and 5 are superimposed.

The effect of the different feedback mechanisms on the regulation of energy supply was examined in numerical simulations using the frequency-change protocol outlined above to increase energy demand. When all feedback mechanisms are active, the increase in pacing frequency leads to a rise in the cytosolic Ca^2+^ amplitude, which brings about an increase in force production and a slight reduction in the amplitude of the membrane potential (Fig.3). The mechanisms responsible for these observations are discussed below. The overall effect on ATP demand is to increase it by approximately three-fold, from 0.3 × 10^−3^ to 1 × 10^−3^ mmol L^−1^ ms^−1^. Figure4 shows the effect of increasing pacing frequency on cytosolic metabolite concentrations (MgATP, MgADP, Pi) and ΔPi/PCr for each of the combinations of feedback mechanisms described above. When all of these feedback mechanisms are inactive (Model 1), the only remaining mechanism in the model to regulate ATP production rate is the underlying substrate availability feedback pathway (ADP and Pi). Therefore, the sensitivity of these metabolite concentrations to the change in energy demand is a measure of the degree to which each of the additional feedback mechanisms regulates ATP production. The results in Figure4 present two distinct sets of curves, which can be grouped as either having Pi-dependent feedback of complex III (Pi-CIII), Models 4 and 5, or not, Models 1–3. Metabolite concentrations for the two feedback mechanisms which increase energy supply by upregulating the dehydrogenase flux (Models 2 and 3) are indistinguishable from the case where all of these feedback mechanisms are inactive and only substrate availability is present (Model 1); MgATP = 5.7 mmol L^−1^, MgADP = 40 *μ*mol L^−1^, Pi = 5 mmol L^−1^ and ΔPi/PCr = 0.32) and hence we conclude that these potential mechanisms do not have a significant influence on regulating ATP production. Stimulation of complex III via Pi feedback, downstream of the dehydrogenase flux, is the only feedback mechanism that reduces the dependence on substrate availability (i.e., the steady-state metabolite concentrations at 3 Hz are lower; MgATP = 5.6 mmol L^−1^, MgADP = 35 *μ*mol L^−1^, Pi = 3 mmol L^−1^ and Pi/PCr = 0.2). This indicates that, of the proposed feedback mechanisms, Pi-dependent feedback of complex III is the only one which can upregulate ATP production rate above that of substrate availability feedback.

**Figure 5:**
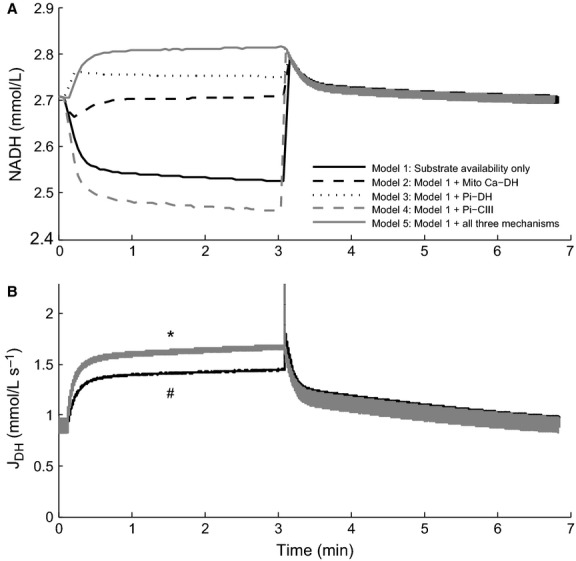
Cell model simulation of the effect of activating different feedback mechanisms on the concentration of mitochondrial NADH (A) and the dehydrogenase flux (B) in response to a change of stimulus frequency from 2 Hz to 3 Hz at *t* = 15 sec and back to 2 Hz at *t* = 3 min. During the 2 Hz simulation period, all three mitochondrial regulatory mechanisms were active. ^#^Models 1, 2 and 3 are superimposed. *Models 4 and 5 are superimposed.

Figure5 shows the effect of the feedback mechanisms on NADH concentration and dehydrogenase flux. Although the different feedback mechanisms give rise to different NADH transients, like the metabolite concentration profiles (Fig.4), there are two distinct dehydrogenase transients, which can be grouped into those with, and those without, Pi-CIII feedback. The dehydrogenase flux is determined by the level of NADH, which is itself determined by demand-driven substrate feedback via complex III and substrate availability. When the dehydrogenase flux is increased independently of NADH (in an open loop manner) by upregulation of the dehydrogenase flux, the NADH concentration responds in such a way as to maintain the flux of ATP required to meet the prevailing energy demand. Thus, cellular energy demand is communicated to the mitochondria only when the feedback pathway directly stimulates processes downstream of the dehydrogenase flux. Examining the case where mechanisms both upstream and downstream of the dehydrogenase flux are active (Model 5), in comparison to downstream regulation (Model 4), shows that, although the dehydrogenase flux transients are the same, the NADH concentrations are higher (2.8 mmol L^−1^ vs. 2.45 mmol L^−1^) when upstream and downstream regulatory mechanisms are present. When the upstream mechanisms are active, they stimulate the dehydrogenase flux above that required by cellular demand. This is buffered by an increase in NADH concentration to down-modulate the dehydrogenase flux to a level that meets the prevailing energy demand. These results suggest that mechanisms which regulate the dehydrogenase flux, which are upstream of the respiratory chain, have relatively little impact on increasing ATP production to meet increased demand. Rather, only direct regulation of complex III in the respiratory chain (by Pi) has any positive effect on ATP production independent of substrate levels, and hence in stabilizing metabolite concentrations. Given that the Pi-CIII mechanism is capable of stimulating ATP production above that of substrate availability feedback to match increases in energy demand, can this mechanism stabilize cell function over a wide range of energy demand?

### Effect of metabolite stability on cell function

The preceding simulations establish that regulation of complex III of the respiratory chain by inorganic phosphate contributes to maintaining relatively stable cytosolic metabolite concentrations, showing significantly lower fluctuations in cytosolic concentrations than was observed when substrate availability was the sole or dominant regulatory feedback on ATP production. Stability of cellular metabolite concentrations is important for maintaining cellular function as the thermodynamic and kinetic properties of cellular ATPases, responsible for ion transport (SERCA and Na^+^/K^+^ pumps) and force production (myosin ATPase), are all dependent on the cellular metabolic state (ΔG_ATP_ and metabolite concentrations). To investigate the effectiveness of Pi-CIII regulation on maintaining cellular function with increasing energy demand, we simulated for 7 min at 3 Hz, at SLs ranging from 1.6 to 2.1 *μ*m.

**Figure 6:**
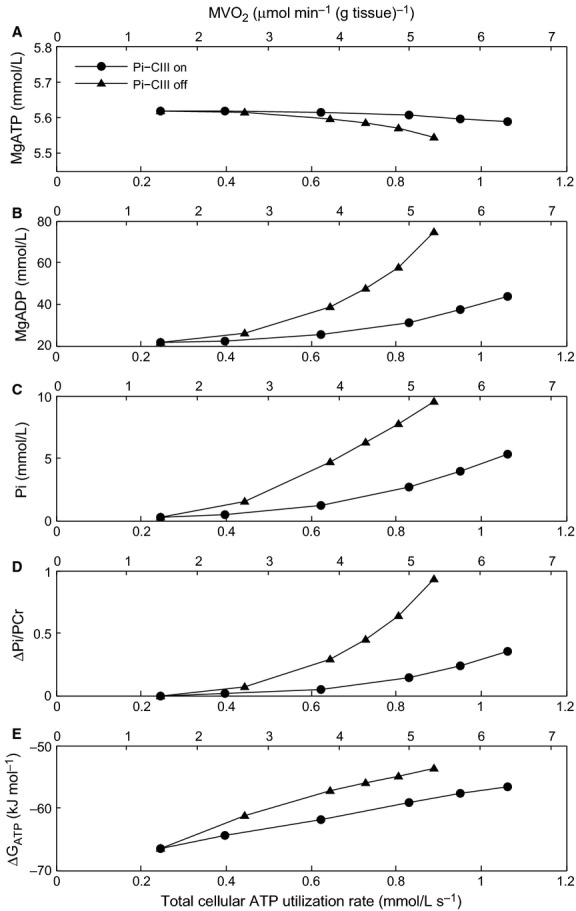
The effect of increasing energy demand, by increasing sarcomere length during isometric contractions, on the concentrations of MgATP (A), MgADP (B) and Pi (C), the ratio of change of Pi to PCr (ΔPi/PCr) (D) and the free energy of MgATP hydrolysis (E). For each panel, the left-most point corresponds to SL = 1.6 *μ*m and the right-most point corresponds to SL = 2.1 *μ*m.

In these simulations, when Pi-CIII regulation is active an increase in energy demand (indexed as ATP utilization rate) leads to smaller changes in cellular metabolite concentrations (Fig.6). While MgATP and MgADP are buffered by PCr, and do not change concentration significantly, in the absence of Pi-CIII regulation, Pi reaches almost 10 mmol L^−1^ at the highest energy demand. Figure6 also shows ATP utilization rescaled in terms of myocardial oxygen consumption (MVO_2_), calculated from the mitochondrial complex IV flux. The MVO_2_ values calculated from the cell model (Fig.6) correspond well with reported experimental values. A maximum MVO_2_ of 10.7 *μ*mol min^−1^ (g tissue)^−1^ has been reported in canine hearts in vivo (Bache et al. 1999; Gong et al. 2003), while a maximum MVO_2_ of 12 *μ*mol min^−1^ (g tissue)^−1^ and 6 *μ*mol min^−1^ (g tissue)^−1^ has been reported in isolated perfused rat and guinea pig hearts, respectively (Cooper et al. 2001). In the case of the guinea pig heart, the maximum MVO_2_ was obtained using potassium chloride (KCl) arrest in the presence of very low extracellular Na^+^ concentrations. This caused the Na^+^/Ca^2+^ exchanger to reverse and the cytosol to flood with Ca^2+^, fully activating the cross-bridges and SERCA pump. The canine data from Zhang and colleagues (Bache et al. 1999; Gong et al. 1999, 2003; Ochiai et al. 2001; Zhang et al. 2003, 2005), which was aggregated and shown in figure 1B of Wu et al. (2009), also indicate that the metabolite concentrations (ΔPi/PCr) remain relatively constant until MVO_2_ is greater than approximately 6 *μ*mol min^−1^ (g tissue)^−1^, before rising rapidly. This compares well with the predicted ΔPi/PCr generated by the model when Pi-CIII regulation is active (Fig.6D). In the absence of Pi-CIII regulation in the model, there is also a reduction in the free energy of ATP hydrolysis (Fig.6E), which reflect the greater increase in the concentrations of ATP hydrolysis products MgADP and Pi and a drop in MgATP.

**Figure 7:**
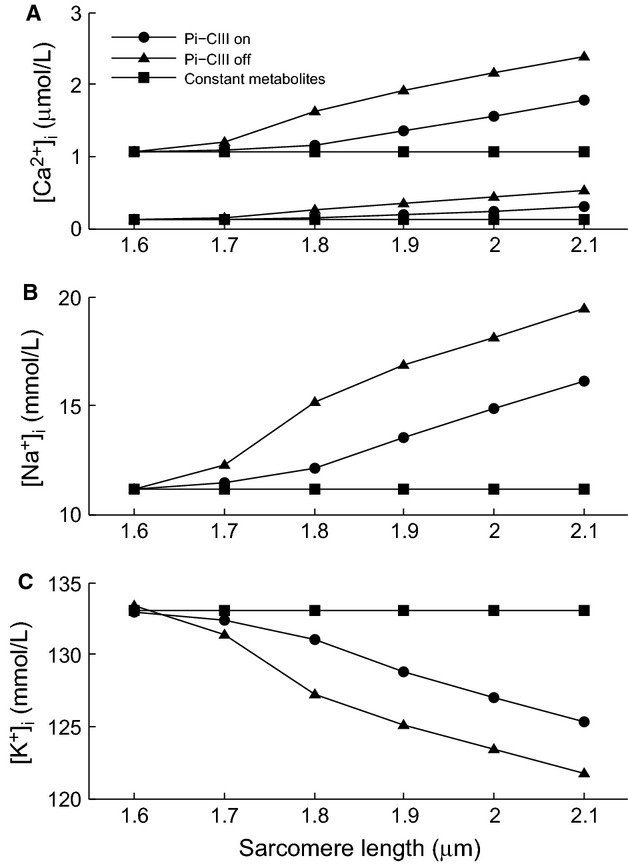
Concentrations of cytosolic Ca^2+^, Na^+^ and K^+^ as functions of increasing sarcomere length, and hence energy demand. Panel A shows the systolic (top 3 traces) and diastolic cytosolic Ca^2+^ concentrations. The ?constant metabolites’ traces refer to simulations where the metabolite concentrations were held constant to mimic the idealized scenario where they are instantly replenished.

The effects of increasing cellular energy demand on cytosolic Ca^2+^, Na^+^, and K^+^ concentrations are shown in Figure7. The “constant metabolite” traces show simulations where the metabolite concentrations are held fixed at constant values. Under this scenario, an increase in ATP utilization rate via stretching of the muscle cell does not lead to changes in the concentration of cytosolic Ca^2+^, Na^+^, or K^+^ and we can therefore conclude that any changes in ion concentrations that are observed under other conditions are not a direct consequence of varying SL. (Note that the effect of length-dependent Ca^2+^ binding to troponin C on the cytosolic free Ca^2+^ has been removed from the model to achieve this.) When metabolite concentrations are allowed to vary dynamically, we observe an increase in cytosolic Na^+^, K^+^, and diastolic and systolic Ca^2+^ with SL (Fig.7). These changes were much more pronounced for the simulation where Pi-CIII regulation was inactive and reflect the greater changes in metabolite concentrations under these conditions.

**Figure 8:**
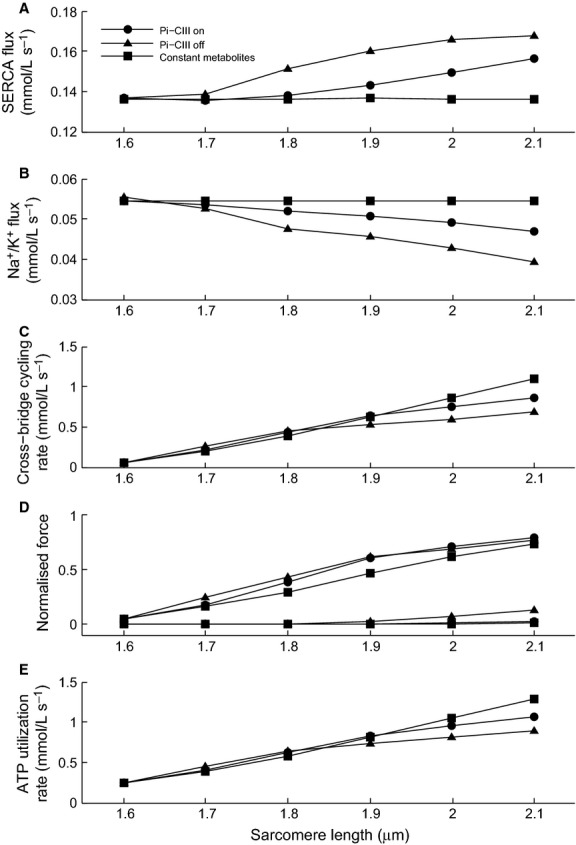
Cycling rates of the SERCA pump (A), Na^+^/K^+^ pump (B), force production (C) and total ATP consumption rate (E) as functions of increasing sarcomere length, and hence, energy demand. The upper and lower sets of traces in panel D represent the systolic and diastolic forces, respectively.

Cytosolic ion concentrations in the cardiomyocyte are determined primarily by SERCA and the Na^+^/K^+^ pump. The change in the metabolic state of the cell as a result of an increase in energy demand leads to opposing effects on SERCA and the Na^+^/K^+^ pump (Fig.8A and B). Relative to simulations with constant metabolite concentrations, SERCA flux is higher when Pi-CIII regulation is active, and increases further when it is absent, for all SLs, while Na^+^/K^+^ pump flux is reduced when Pi-CIII regulation is active, and decreases further when it is absent. The Na^+^/K^+^ pump flux is reduced since the enzyme is inhibited by increasing MgADP and Pi and reduced pH (data not shown), which leads to the rise in the cytosolic Na^+^ concentration. As a result, the Na^+^/Ca^2+^ exchanger is driven in the reverse, which elevates cytosolic Ca^2+^. The acidification of the cell also contributes to the rise in cytosolic Na^+^ by stimulating the Na^+^/H^+^ exchanger. The changing metabolite concentrations also have an inhibitory effect on the kinetics of the SERCA pump (Tran et al. 2009), but inhibition was overcome by the large increase in cytosolic Ca^2+^ concentration. The presence of Pi-CIII regulation mitigated the inhibition of both the SERCA and the Na^+^/K^+^ pumps and, as such, was able to maintain the fluctuation in cytosolic ion concentrations in response to increased energy demand.

The above changes also have direct consequences for force generation. In the absence of Pi-CIII regulation, the myosin ATPase rate is lower for a given SL, indicating reduced cross-bridge cycling rate due to the inhibitory effect of rising Pi (Tran et al. 2010) (Fig.8C). Increased diastolic Ca^2+^ (Fig.7A) also elevates the diastolic force, and compromises the ability of the myocyte to relax completely (Fig.8D). These results indicate that maintenance of stable metabolite concentrations is necessary for cytosolic ion homeostasis and mechanical force output and that this is promoted through Pi regulation of respiratory complex III.

## Discussion

The regulation of cardiac energy supply in response to changing demand is critically important in ensuring normal cardiac function. The mechanisms underlying this regulation are not yet fully understood, although a number of hypotheses have been advanced. In this study, we have developed a mathematical model of cardiac ventricular cell function to investigate the effect of different possible mechanisms in regulating cardiac energy supply. The model couples thermodynamic formulations of metabolite-sensitive models of the energy-demand processes: SERCA (Tran et al. 2009); Na^+^/K^+^ ATPase (Terkildsen et al. 2007); and cross-bridge myosin-ATPase (Tran et al. 2010), to a model of mitochondrial oxidative phosphorylation (Beard 2005), within an electrophysiological framework (Crampin and Smith 2006).

We find that the intrinsic substrate availability pathway (availability of ADP and Pi for ATP resynthesis) alone is capable of upregulating mitochondrial ATP production to meet increased energy demand. We find that neither Ca^2+^- nor Pi-mediated regulation of the dehydrogenase flux is capable of upregulating mitochondrial ATP production rate above that of substrate availability (from MgADP and Pi) (Fig.4). In striking contrast, we find that Pi-dependent activation of mitochondrial respiratory complex III can contribute to upregulation of mitochondrial ATP production. Furthermore, we find that this activation of complex III, downstream of the dehydrogenase flux, leads to increased stability of cellular metabolite concentrations: the implications of this are discussed further below.

Although many studies have reported an effect of Ca^2+^ on dehydrogenase activity or respiration rate (Denton et al. 1978; Kobayashi and Neely 1983; Hansford 1987; McCormack et al. 1990; Harris and Das 1991), this is not entirely inconsistent with our model results. While activation of dehydrogenase flux by Ca^2+^ or Pi appears to have no impact on increasing ATP production rate over and above that of substrate availability, it does impact on the NADH concentration, which is an indicator of mitochondrial ATP production *capacity*. When dehydrogenase stimulation is activated (Fig.5), there is a rise in the steady-state concentration of NADH, increasing the capacity for respiration. However, in our simulations of an intact cell under physiological conditions, this capacity is never reached because the limiting factor is the availability of ADP and Pi, rather than the dehydrogenase flux. In contrast, in vitro experiments on isolated mitochondria are carried out under conditions where the availability of ADP and Pi are not limited (state 3), in order to measure the maximal respiration rate. Under these conditions, the limiting factor is the dehydrogenase flux. Baniene et al. (2006) have reported an increase in mitochondrial respiration rate in response to an increase in Ca^2+^ in mitochondria isolated from guinea pig hearts at 28°C and 37°C under state 3 respiration. The increase in maximal respiration rate is in accordance with our model predictions with regard to increase in respiratory capacity, which can be realized only when substrate availability is not a limiting factor. Our simulations indicate that the physiological level of ATP utilization and the associated availability of substrates are well below the threshold where dehydrogenase flux becomes the limiting factor. Activation of dehydrogenase fluxes therefore cannot increase respiration rate under physiological conditions. In support of this conclusion, Ochiai et al. (2001), in their study of the blood-perfused intact dog heart, reported that supplementation of supraphysiological levels of pyruvate did not increase the rate of MVO_2_ during a high work state, achieved by dobutamine and dopamine infusion. They concluded that the availability of carbohydrate substrates (to fuel the dehydrogenase fluxes) is not a limiting factor for ATP synthesis. Furthermore, supplementation of pyruvate had no significant effect on reducing ΔPi/PCr during the high work state, consistent with the effect of dehydrogenase activation in our model (Fig.4). In the mitochondrial model used here, the maximum dehydrogenase flux, *X*_DH_, was originally set quite high (Beard 2005), such that NADH did not fall when energy demand was increased. It was therefore reduced in order to observe a fall in NADH concentration with increasing frequency to match data from Brandes and Bers (2002).

Our model predicts that NADH concentration is not a good indicator of mitochondrial respiration rate. For a given dehydrogenase flux, there can be multiple NADH values (Fig.5). Activation of the dehydrogenase flux by Pi or Ca^2+^ is countered by a shift in the concentrations of NADH and NAD^+^ such that the dehydrogenase flux remains unchanged and matches the rate of ATP demand signaled by substrate availability. Contrasting experimental results on the effect of work rate on NADH levels implicitly support this notion. NADH concentration has been shown to decrease with increased work in rat ventricular myocytes (White and Wittenberg 1993, 1995), whereas the opposite was seen by Katz et al. (1987) and Koretsky and Balaban (1987) in the isolated whole heart.

Of these three proposed mechanisms, our simulations suggest that the only one capable of increasing mitochondrial ATP supply above that of substrate availability, is Pi regulation of respiratory complex III. This mechanism was proposed by Beard (2005) as the only one (out of 19 other possible mechanisms) capable of producing a good fit of their mitochondrial model to data from Bose et al. (2003) on mitochondrial membrane potential as a function of buffer Pi at two different ADP concentrations. The other 18 mechanisms tested the effect of other species (ATP, ADP, Mg^2+^) as controllers of complexes I, III, and IV and of F_1_F_0_ ATPase and the ANT system and were all excluded on the basis that they were not able to reproduce the observed data. We have considered this mechanism, originally identified based on the data of Bose et al. (2003), in the context of cellular feedback on mitochondrial respiration, where metabolite and ion concentrations are dynamically changing, and have shown that it is capable of reducing the fluctuations of metabolites and consequently stabilizing cellular ion concentrations in response to increasing energy demand.

### Cytosolic ion concentrations and cell function

Our simulations have demonstrated that changes in the concentrations of ADP and Pi are necessary for communicating the changes in energy demand to modulate mitochondrial ATP production, through the intrinsic feedback pathway of substrate availability. The rise in ADP and Pi in response to an increase in energy demand is a result of the increased rate of hydrolysis of ATP by the SERCA and Na^+^/K^+^ pumps as well as the myosin ATPase. Fuelled by the hydrolysis potential, the two pumps consume ATP thereby maintaining cytosolic Ca^2+^, Na^+^ and K^+^ concentrations while the myosin ATPase converts the chemical energy to force production. These energy-demand processes are, however, also inhibited by rising ADP and Pi. Hence, the regulation of mitochondrial energy supply, which determines cytosolic metabolite concentrations (Fig.4) is an important determinant of cellular function. When Pi-CIII regulation is active, the upregulation of mitochondrial ATP production reduces the extent to which the metabolite concentrations change (Fig.6). As a result, the effect on the cycling of the Na^+^/K^+^, SERCA and myosin ATPases are not as great (Fig.8) and the cytosolic concentrations of Na^+^, Ca^2+^, and K^+^ are maintained within a narrower range (Fig.7). The change in cytosolic ion concentrations is a result of a cascading series of events, initiated by the slowing of Na^+^/K^+^ pump kinetics by the hydrolysis products MgADP and Pi, which leads to a build-up of Na^+^ and a drop in K^+^. The high Na^+^ reduces the Ca^2+^ efflux by the Na/Ca exchanger, leading to increases in the systolic and diastolic Ca^2+^ levels. The rise of cytosolic Na^+^ concentration, as a result of Na^+^/K^+^ pump inhibition, is consistent with studies on quiescent guinea pig myocytes, which have reported a rise in cytosolic Na^+^ from 6.6 to 20.1 mmol L^−1^ after the addition of strophanthidin, a cardiac glycoside that inhibits Na^+^/K^+^ pump activity. This was accompanied by a rise in the cytosolic Ca^2+^ concentration (Satoh et al. 1994). The rise in Na^+^ and Ca^2+^ is a consequence of the imperfect matching of energy supply to demand, which, qualitatively, reflects the effects seen in ischemic myocytes, where a lack of energy supply leads to elevated Na^+^ and Ca^2+^ concentrations (Nakamura et al. 1999). There is an overall null effect on systolic force production because the inhibitory effect of rising ADP and Pi on cross-bridge force production is mitigated by the elevated systolic Ca^2+^ concentration. However, when Pi-CIII regulation is inactivated, the greater rise in diastolic Ca^2+^ (Fig.7A) and greater slowing of cross-bridge cycling rate (Fig.8C) may signal the onset of diastolic heart failure as indicated by an elevation of the diastolic force (Fig.8D). Overall, the simulations predict that Pi-CIII regulation improves the coupling between energy demand and supply, above that of substrate feedback, and maintains the ion concentrations within a tighter range.

### Metabolic stability hypothesis

The changes in metabolite concentrations predicted by our model contradict the metabolic stability hypothesis of Balaban and co-workers (Balaban et al. 1986; Katz et al. 1989) who concluded that metabolite values remain constant over a wide range of workload (Fig.6) and hence cannot determine the ATP production rate. Our model predicts that for low ATP utilization rates (below 0.6 × 10^−3^ mmol L^−1^ ms^−1^), the steady-state metabolite concentrations change very little, but above that, they do start to change by an appreciable amount (Wu et al. 2008; Beard and Kushmerick 2009). The ΔPi/PCr predicted by the model (Fig.6D) is consistent with the simulations and experimental data shown in figure 1B of Wu et al. (2009). We have demonstrated that these metabolite changes are necessary and sufficient to communicate increases in demand via the substrate availability and Pi-CIII pathways to the mitochondrial oxidative phosphorylation system in order to maintain cellular force production and cytosolic ion homeostasis.
